# The association between exacerbation of chronic obstructive pulmonary disease and timing of paracetamol use: a cohort study in elderly Australians

**DOI:** 10.1186/s12931-022-02010-z

**Published:** 2022-04-05

**Authors:** Thu-Lan Kelly, Michael Ward, Nicole L. Pratt, Emmae Ramsay, Marianne Gillam, Elizabeth E. Roughead

**Affiliations:** 1 Clinical and Health Sciences, Quality Use of Medicines Pharmacy Research Centre, University of South Australia, Adelaide, Australia; 2 Clinical and Health Sciences, University of South Australia, Adelaide, Australia

**Keywords:** Chronic obstructive pulmonary disease, Paracetamol, Weighted cumulative exposure, Medicine safety

## Abstract

**Background:**

In elderly populations, paracetamol may be used regularly for conditions such as osteoarthritis. Paracetamol has been associated with respiratory disease through a proposed mechanism of glutathione depletion and oxidative stress. Given that chronic obstructive pulmonary disease (COPD) is frequently co-morbid with osteoarthritis, this study investigated whether the dose and timing of paracetamol exposure may induce COPD exacerbations.

**Methods:**

The study population was 3523 Australian Government Department of Veterans’ Affairs full entitlement holders who had existing COPD on 1 January 2011, who were dispensed at least one prescription of paracetamol between 1 January 2011 and 30 September 2015, and had no paracetamol dispensed in the 6 months prior to 1 January 2011. The outcome was time to first hospitalisation for COPD exacerbation after initiation of paracetamol. A weighted cumulative exposure approach was used.

**Results:**

The association between paracetamol exposure and COPD exacerbation was protective or harmful depending on the dose, duration, and recency of exposure. Compared to non-use, current use at the maximum dose of 4 g daily for 7 days was associated with a lower risk (HR = 0.78, 95% CI = 0.67–0.92) and a higher risk after 30 days (HR = 1.27, 95% CI = 1.06–1.52). Risk declined to baseline after 2 months. For past use, there was a short-term increase in risk on discontinuation depending of dose, duration and time since stopping.

**Conclusions:**

Patients and doctors should be aware of the possible risk of COPD exacerbation with higher dose paracetamol 1 to 6 weeks after initiation or discontinuation, but no increased risk after 2 months.

**Supplementary Information:**

The online version contains supplementary material available at 10.1186/s12931-022-02010-z.

## Background

Paracetamol, or acetaminophen, is the most commonly used analgesic worldwide and also has antipyretic properties. Paracetamol is first-line treatment for osteoarthritis, estimated to affect 300 million people worldwide [1].

In recent years, paracetamol has been associated with the development or exacerbation of asthma in children and adults. Studies of paracetamol use in adults and children have shown a relationship between frequency of use of paracetamol and asthma exacerbations, with use of higher doses or longer duration of exposure associated with higher estimates of risk [2–6]. A systematic review and meta-analysis [7] found that any paracetamol exposure in the first trimester of pregnancy was associated with an increased risk of childhood asthma in those exposed in-utero (OR = 1.39, 95% CI 1.01–1.91). However, the association was highly variable between studies and only one study adjusted for maternal respiratory tract infections.

The proposed mechanism by which paracetamol is associated with asthma exacerbation is through depletion of lung glutathione and subsequent oxidative stress, which is pro-inflammatory and increases bronchoconstriction [8, 9]. This response appears to be dose-dependent, with animal models showing higher doses of paracetamol associated with more depletion of lung glutathione [10, 11]. One study showed baseline rat lung glutathione levels took 8 days to return to baseline after exposure to a single supratherapeutic dose of paracetamol of 3 g/kg [11]. Oxidative stress is considered to be one of the major drivers of Chronic Obstructive Pulmonary Disease (COPD) [12], particularly during exacerbations where significant depletion of gluthathione and increased markers of oxidative stress have been observed [13]. There is evidence that the ‘lung glutathione depletion’ hypothesis may apply to COPD as studies have found that *N*-acetylcysteine, a source of building blocks for intracellular glutathione, improved lung function and reduced exacerbations in patients with COPD [14] but it is unclear whether these effects are due to mucolytic actions or through modification of oxidative pathways [15]. McKeever et al. [16] found persons with a COPD diagnosis were more likely to use paracetamol than those without. This study also found that people with COPD who used paracetamol daily had decreased lung function, with a lower mean adjusted forced expiratory volume of 61.5 ml (95% CI − 97.5 to − 25.4) compared with nonusers.

Paracetamol is used extensively in the elderly population, where the burden of COPD is high [17]. COPD is the leading cause of avoidable hospitalization in Australia [18, 19]. If regular paracetamol use, such as for osteoarthritis, is a contributing factor towards COPD exacerbations, then recommendations to reduce paracetamol daily doses to the minimum required to reduce pain would potentially prevent many hospitalizations.

The aim of our study was to investigate the relationship between the dose and timing of paracetamol exposure and hospitalization for COPD exacerbation. The association between paracetamol and COPD exacerbation, if present, is likely to be complex and we hypothesized, based on previous research in asthma, that the effect is likely to be cumulative and dose dependent. Medicine exposure is conventionally classified using broad categories such as ‘daily, ‘current’ or ‘recent’, however this may not be sufficient to capture a more complex relationship between paracetamol and COPD exacerbation. Therefore, we used the innovative weighted cumulative exposure method [20] which combines dose, duration and recency of exposure to predict risk of an outcome.

## Methods

### Study population

The data for this study were obtained from the Australian Government Department of Veterans’ Affairs (DVA) administrative claims database. This database contains records of all prescription medicines dispensed under the Pharmaceutical Benefits Scheme and Repatriation Pharmaceutical Benefits Scheme and paid for which DVA pay a subsidy, as well as all medical, allied health services and hospitalizations provided to subjects for whom DVA pays a subsidy.

### Study cohort

**Fig. 1:**
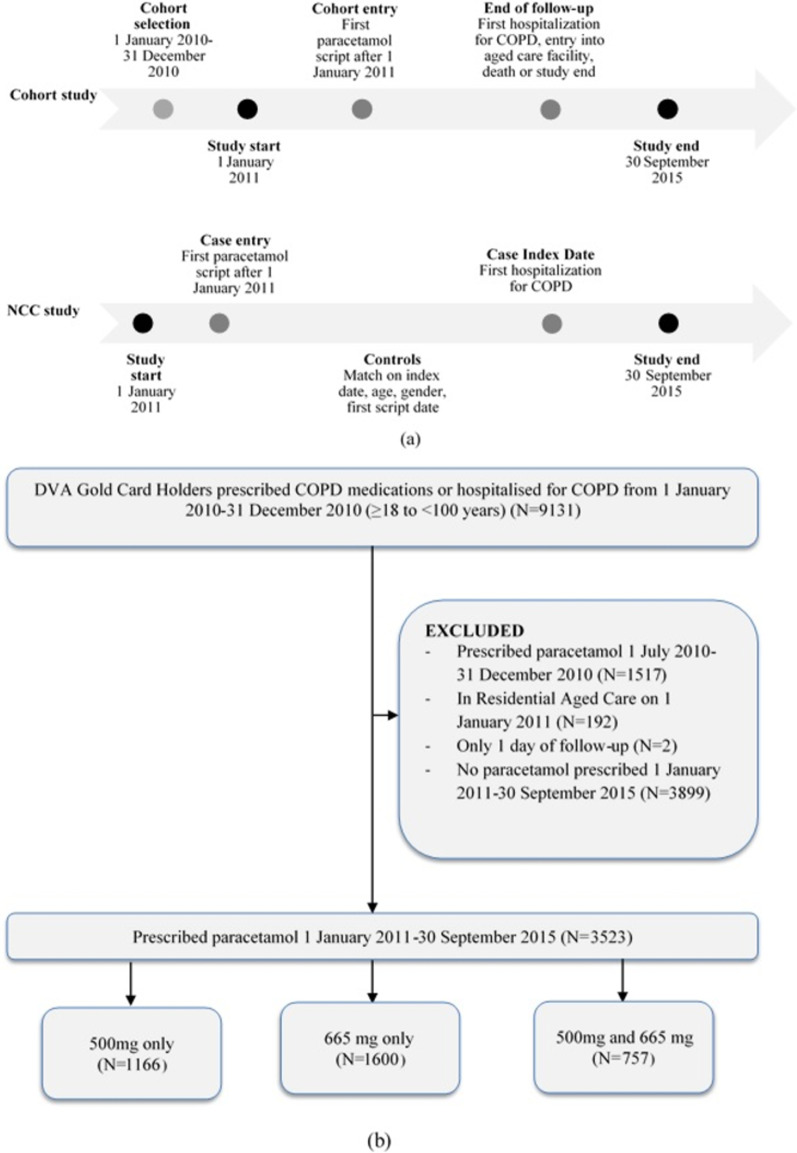
**a** Timeline of study design for cohort and nested case–control (NCC) study. **b** Flow chart of COPD cohort selection

Figure 1a shows the timeline of the study design. The study period was 1 January 2011–30 September 2015. Paracetamol was available on prescription during this period and the majority of use was likely to be by prescription because it was less expensive for the cohort due to a subsidy. Patients were eligible for inclusion in the study if on 1 January 2011 (the study start) they were aged between 45 and 100 years, and in the previous 12 months they were full entitlement holders and had existing COPD (N = 9131, Fig. 1b). Evidence of COPD at the study start was defined by dispensing of at least one medicine indicated for COPD (ATC code R03BB, excluding R03BB01) or at least one hospital admission with a recorded diagnosis of COPD (primary or secondary ICD-10-AM Diagnosis code J43*, J440, J441, J448, J449) in the 12 months prior to study start. The ICD-10 codes represent hospitalization for emphysema or other chronic obstructive pulmonary disease, but exclude hospitalization for bronchitis only.

Patients were excluded if they were dispensed any paracetamol (ATC Code N02BE01, see Additional file 3) in the 6 months prior to study start (1517 excluded), were in residential aged care at study start (192 excluded), or had only 1 day of follow-up (2 excluded).

Since the population of interest was COPD patients who were chronically exposed to paracetamol, such as those co-morbid with osteoarthritis, 3899 patients who did not initiate paracetamol during the study period were excluded. Patients who had no dispensings of paracetamol were likely to have been exposed during follow-up through over the counter products and their inclusion may have produced misclassification bias. The final cohort consisted of 3523 patients with existing COPD who had been dispensed a least one paracetamol script during the study period. Following the approach of Sylvestre et al. [21] to avoid immortal time bias, patients entered into the study (cohort entry) on the date the first paracetamol script was dispensed after the study start.

### Exposure assessment

Each patient’s individual history of paracetamol use was based on prescriptions dispensed throughout the study period, starting from initiation until the day before the end of follow-up. All prescriptions dispensed for paracetamol, including 500 mg and controlled release 665 mg tablets were included. No patients in the study cohort were dispensed the paracetamol/codeine combination. If prescriptions were re-filled before the end of the current supply period, the extra days of supply were added to the end of the current supply period to extend exposure. Daily dosage was calculated assuming the maximum dose of paracetamol (3.99 or 4 g/day) was used each day given the majority of use in this cohort is likely to represent regular use associated with treating pain of osteoarthritis. Co-morbid osteoarthritis was determined using either hospitalization with a primary or secondary diagnosis of osteoarthritis (ICD-10-AM codes M15*–M19*) during the baseline period or after the study start, or dispensed at least one script of controlled release paracetamol during follow-up.

### Outcome assessment

The outcome was defined as any admission to hospital after cohort entry (i.e. initiation of paracetamol) with a primary ICD-10 diagnosis code of COPD exacerbation (J43*, J440, J441, J448, J449) i.e. excluding hospitalization primarily for bronchitis. Follow-up time was determined from paracetamol initiation until COPD exacerbation, death, entry to RAC or study end (30 September 2015), whichever was the earliest.

### Assessment of covariates

Factors known to influence the risk of COPD in the literature, including age, sex, hypertension, congestive heart failure and diabetes, number of comorbidities (excluding the three specified) and use of statins [17] were selected as covariates for inclusion into the models. Number of and specific co-morbidities were determined using the Rx-Risk definitions and score [22].

### Statistical analysis

#### Medication exposure modeling

To assess the relationship between paracetamol dose and duration of use with COPD exacerbation, we used the weighted cumulative exposure (WCE) method described by Sylvestre et al. [20]. The WCE model uses information on past exposures including duration, dose and timing of exposure, to estimate the risk of the adverse event. The time-varying WCE models weight each dose by the relative importance of that dose at that time on the current risk of the adverse event under study.

Paracetamol exposures were modelled as the weighted sum of past doses. As the form of the association between cumulative doses of paracetamol and COPD exacerbation was unknown, we used a flexible WCE cubic spline model to estimate the weight function directly from the data [20]. SAS version 9.4 (SAS Foundation, Cary, USA) and R version 3.5.1 (R Foundation for Statistical Computing, Vienna, Austria) were used for statistical analyses.

#### Survival models

Using the full cohort (N = 3523), weights were calculated using Cox proportional hazards models to model time to COPD exacerbation with flexible cubic splines fitted to the daily dose. Censoring occurred at death, entrance into an aged-care facility, or the end of study. Follow-up was the number of days between cohort entry and hospitalization for COPD or censoring. Age, sex and co-morbidities in the 12 months before cohort entry were included as fixed in time covariates, while statin use, defined as a binary indicator of exposure for every day during follow-up, was included as a time-varying covariate. Models were stratified by disease severity, defined as any hospitalization for COPD or systemic glucocorticoids (ATC: H02AB) use in the 12 months before the study start (baseline period). The ‘WCE’ R package [23] (version 1.02) was modified to allow for stratified Cox models. The proportionality assumption in the Cox models was tested using Schoenfeld residuals for all the covariates and the fitted splines.

#### Nested case–control study

To confirm the results of the cohort study, in particular to compare the best fitting weight function and the size of the time window from the WCE method, a nested case–control (NCC) study was conducted with cases and controls selected from the same cohort. The cases were all patients who experienced hospitalization for COPD exacerbation (N = 619) and the index date was the date of hospitalization. Two controls per case who had not experienced the outcome on the corresponding index date were matched on sex, age at index date (within 2 years) and the date of the first paracetamol prescription dispensing (within 30 days) in the study period. Controls may have been used more than once and may have become a case later. Matching was not possible for 25 cases who were excluded from the analysis, resulting in 594 cases and1188 controls.

Statistical analysis was performed with the nested case–control version of the weighted cumulative exposure method using conditional logistic regression models [24]. Models were adjusted for co-morbidities described previously in the 12 months before the index date, any statin use in the 30 days before the index date and the number of health services used in the 12 months before the index date (log-transformed) as a proxy for disease severity.

#### Conventional models

We compared the WCE models with conventional models. In survival analysis, conventional models may classify time varying exposure as current use, where exposure is defined as a binary indicator for each day of follow-up; or current dose, with exposure defined as the dose on each day of follow-up. Since we calculated the daily dose as the maximum allowable per day, these models differ only by a scale factor (a binary indicator of 1 was equivalent to the maximum dose) and therefore we included current dose models only. We also investigated other exposure definitions such as mean or cumulative dose in the 2 to 90 days before the current follow-up day.

For the nested case–control study, we used conditional logistic regression models, with fixed-in-time exposure modelled as mean or any (binary) exposure in the 7, 15, 30, 60, 75 and 90 days before the index date.

## Results

Descriptive characteristics of the cohort study are presented in Table 1 and the nested case–control study in Table 2. Overall, of 3523 patients who were dispensed paracetamol during the study period, the median age was 85 years and 1339 (38%) were female. Patients were exposed to paracetamol for a median 25% of their follow-up time and chronic users with at least 60 days of exposure were 64% of the cohort. Osteoarthritis was co-morbid in 2453 patients (70%). 619 (18%) patients were hospitalized with a primary diagnosis of COPD during follow-up.

**Table 1:** Descriptive statistics of the cohort study

Characteristic	All patients
N	3523
Age at cohort entry, median [IQR]	85 [78–89]
Female sex, n (%)	1339 (38.0)
Rx-risk comorbidity score^a,b^, median [IQR]	5 [3–6]
Hypertension^a^ n (%)	1598 (45.4)
Congestive heart failure^a^ n (%)	809 (23.0)
Diabetes^a^ n (%)	442 (12.5)
Co-morbid osteoarthritis, n (%)	2453 (69.6)
Controlled release paracetamol, n (%)	2357 (66.9)
Hospitalization with osteoarthritis, n (%)	410 (11.6)
High baseline COPD severity^c^, n (%)	1364 (38.7)
Prior oral cortisone use^a^, n (%)	1062 (30.1)
One or more prior COPD hospitalizations^a^, n (%)	562 (16.0)
Any statin use during follow-up, n (%)	1883 (53.4)
Hospitalization for respiratory infection at end of follow-up, n (%)	189 (5.4)
Days exposed, median [IQR]	99 [33–283]
Proportion of time exposed during follow up, median [IQR]	0.254 [0.097–0.565]
Chronic users^d^, n (%)	2245 (63.7%)
Follow up (years), median [IQR]	1.98 [0.72–3.43]
COPD hospitalization during follow up, n (%)	619 (17.6%)

^a^In the 12 months before cohort entry

^b^Excluding hypertension, congestive heart failure, diabetes and COPD

^c^Defined as hospitalization for COPD or cortisone use in the 12 months before cohort entry

^d^≥ 60 days exposure

**Table 2:** Descriptive statistics of the case control study

Characteristic	Cases	Controls	p-value
N	594	1188	
Age at index date, median [IQR]	86.5 [80–89]	87 [81–90]	0.95
Sex Female, n (%)	203 (34.2)	406 (34.2)	–
Rx-risk comorbidity score^a,b^, median [IQR]	7 [5–8]	6 [5–8]	< 0.001
Hypertension^a^, n (%)	241 (40.6)	544 (45.8)	0.04
Congestive heart failure^a^, n (%)	168 (28.3)	267 (22.5)	0.008
Diabetes^a^, n (%)	62 (10.4)	121 (10.2)	0.93
Co-morbid osteoarthritis, n (%)	403 (67.9)	859 (72.3)	0.053
Controlled release paracetamol, n (%)	383 (64.5)	829 (69.8)	0.03
Hospitalization with osteoarthritis, n (%)	66 (11.1)	124 (10.4)	0.68
Number of health service visits^a^, median [IQR]	50 [32–74]	43 [27–61]	< 0.001
Statin use^c^, n (%)	274 (46.3)	615 (51.8)	0.032
Days exposed, median [IQR]	66 [33–153]	66 [33–158]	0.33
Proportion of time exposed during follow up, median [IQR]	0.276 [0.120–0.564]	0.264 [0.114–0.542]	0.35
Chronic users^d^, n (%)	332 (55.9%)	633 (53.3%)	0.32
Follow up (years), median [IQR]	0.99 [0.43–2.05]	1.00 [0.43–2.06]	0.99

^a^In the 12 months before the index date

^b^Excluding hypertension, congestive heart failure, diabetes and COPD

^c^Any statin use in the 30 days before the index date

^d^≥ 60 days exposure

In the nested case–control study, cases and controls were balanced on the matching variables age, sex, paracetamol exposure time and follow-up time as well as diabetes in the 12 months before the index date. There was no difference in the proportion of chronic paracetamol users. However, hypertension in the 12 months before the index date and statin use in the 30 days before the index date was lower in the cases than in the controls, while congestive heart failure, the remaining number of co-morbidities and the number of health service visits were higher in the cases. Slightly more controls were dispensed controlled release paracetamol, but there was no difference in co-morbid osteoarthritis between case and controls.

### Effect of paracetamol use on risk of COPD exacerbation

**Fig. 2:**
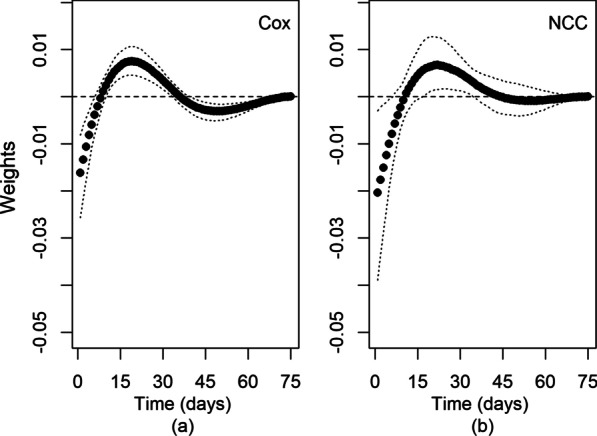
Best fitting weight functions with **a** survival (Cox) models for cohort and **b** nested case control (NCC) studies. Dotted lines indicate confidence bands

The shape of the weight function for the cohort and nested case–control studies was similar (Fig. 2). In both studies, using a flexible spline function with one knot and a time window of 75 days produced the best fitting weight function. In the survival model, time zero in the plot of the weight function is the current day of follow-up, and time on the x-axis corresponds to days before the current day of follow-up (Fig. 2a). In the nested case–control study, time zero is the index date and time on the x-axis corresponds to days before the index date (Fig. 2b). Confidence intervals were wider in the nested case–control version, particularly for time close to zero.

The WCE survival model was the best fitting model of the WCE and conventional models for the cohort and nested case–control studies. More information on the model fit comparisons is found in Additional file 3: Table S1.

**Fig. 3:**
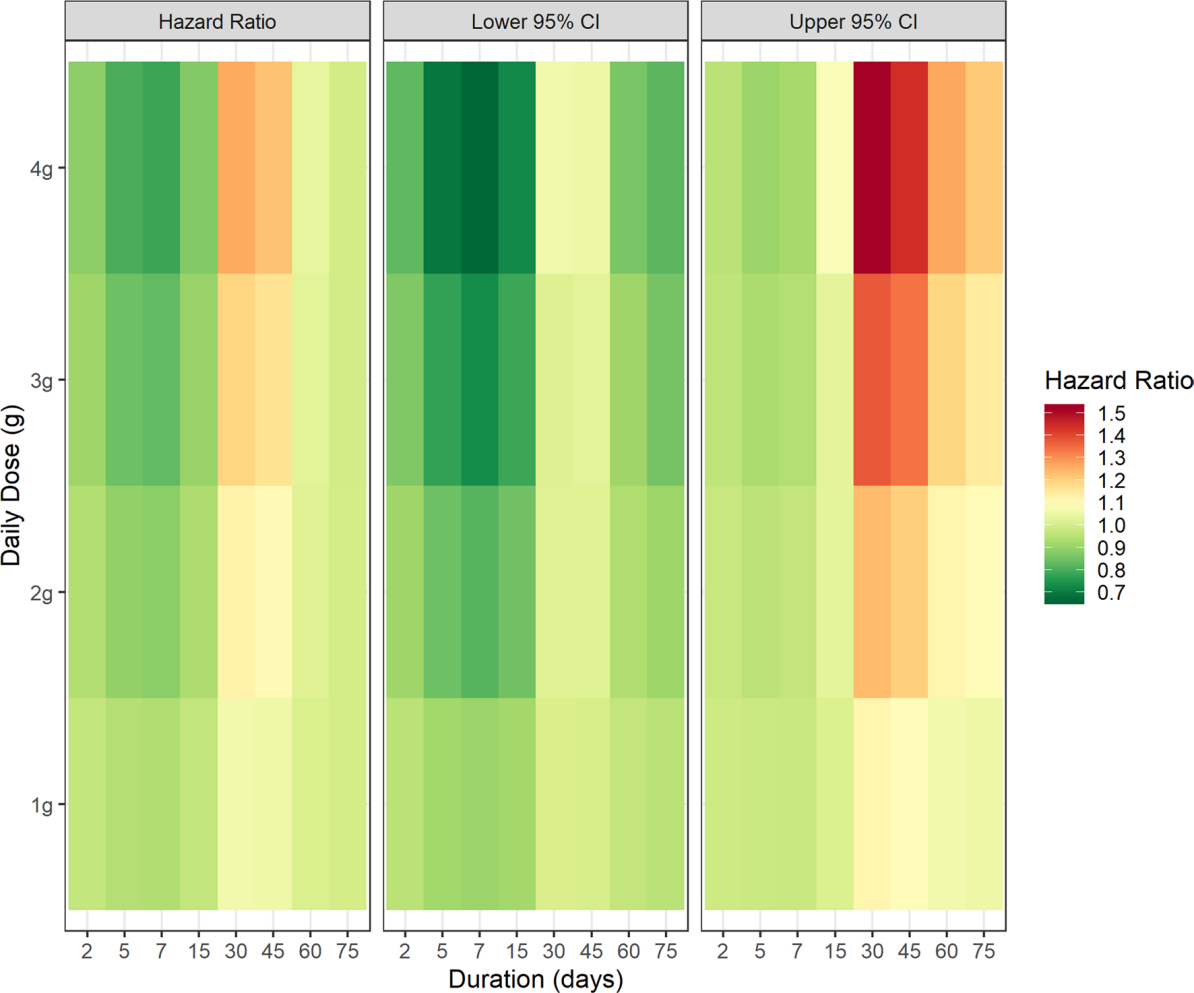
Adjusted Hazard Ratios for different patterns of cumulative paracetamol dose and duration of exposure for 1–4 g daily (compared to non-use)

Table 3 shows the Hazard Ratio (HR) estimates for the covariates included in the WCE survival model. Age, sex, statin use, hypertension assessed by medication use and number of co-morbidities were significant risk factors for COPD exacerbation. An advantage of the WCE approach is that once the shape of the weight function has been determined, the model can be used to predict risk for different exposure patterns and for different comparison groups [20]. In Fig. 2a, negative weights for the WCE survival model in the first week after time zero suggest a small transient protective effect in current users after initiation and a short-term increase in risk after discontinuation [25, 26]. Figure 3 is heatmap of COPD hospitalization rate for different patterns of current paracetamol use compared with non-users. Due to the negative weights, there is a reduced rate of hospitalization for COPD after 2 to 7 days of continuous use (HR = 0.89, 95% CI = 0.83–0.96 after 4 g for 2 days; HR = 0.78, 95% CI = 0.67–0.92 after 4 g for 7 days). Conversely, positive weights 1 to 6 weeks after commencing paracetamol resulted in an increased rate of COPD exacerbation compared with non-use, with the highest rate after 30 days of exposure (HR = 1.27, 95% CI = 1.06–1.52 after 4 g daily for 30 days; HR = 1.23, 95% CI = 1.05–1.44 after 4 g daily for 45 days). After 7 to 8 weeks, the weights in Fig. 2a were close to zero, which may be interpreted as no increase in risk from cumulative exposure after approximately 2 months (4 g daily after 60 days, HR = 1.04, 95% CI = 0.86–1.27). Additional file 1: Figure S1 shows hazard ratios and 95% CIs for current cumulative use of 4 g daily for up to 75 days.

**Table 3:** Covariate estimates from WCE survival model

Variable	Comparison	Adjusted hazard ratio (95% CI)	p-value
Age at cohort entry	Every 1 year increase	1.03 (1.01, 1.03)	< 0.001
Sex	Female vs. male	0.75 (0.63, 0.88)	0.0005
Statin use during follow-up	Any use vs. no use	0.83 (0.71, 0.99)	0.04
Hypertension^a^	Yes vs. no	0.80 (0.67, 0.94)	0.02
Congestive heart failure^a^	Yes vs. no	1.27 (1.05, 1.53)	0.07
Diabetes^a^	Yes vs. no	0.89 (0.69, 1.16)	0.23
Rx-risk co-morbidity score^a,b^	Every extra co-morbidity	1.06 (1.02, 1.09)	0.003

^a^Based on medication use in the 12 months before cohort entry

^b^Excluding hypertension, congestive heart failure, diabetes and COPD

**Fig. 4:**
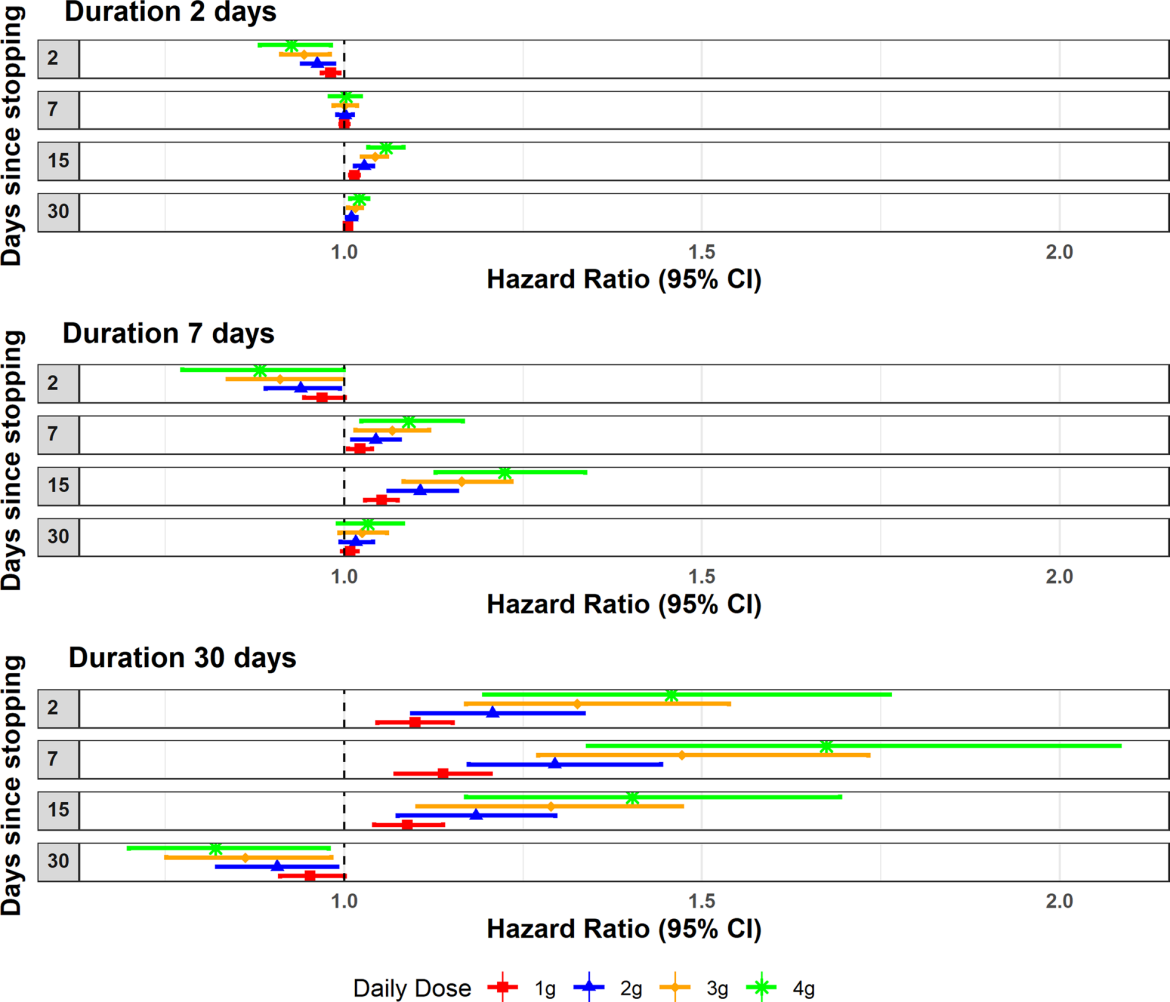
Adjusted Hazard Ratios for past use of 1–4 g daily taken for 2, 7 and 30 days compared to non-use by days after stopping paracetamol

After stopping paracetamol, the effect of paracetamol varied between protective and harmful, depending on dose, duration and time since stopping (Fig. 4). For example, the HR (95% CI) from exposure to 4 g of paracetamol taken for 30 days, after stopping for 2, 7 and 30 days was HR = 1.46 (1.20–1.76), 1.67 (1.34–2.08) and 0.82 (0.70–0.98), respectively. Approximately 2 months after stopping prolonged paracetamol use of 4 g for 75 days, risk returned to baseline (HR = 0.99, 95% CI = 0.98–1.00, 66 days after stopping 4 g of paracetamol used daily for 75 days) (Additional file 2: Fig. S2).

## Discussion

In this study, we found that the rate of COPD exacerbation varied with timing and duration of exposure to paracetamol. There was a decreased rate of COPD exacerbation in the first week after initiation of paracetamol, which changed to a small increased rate after approximately 4 to 6 weeks of continuous paracetamol use (HR upper 95% CI < 1.6 for 4 g daily). However, the rate decreased to baseline levels for durations longer than 2 months. There was also a short-term increased rate after stopping paracetamol, which was dependent on dose, duration and time since stopping (HR upper 95% CI < 2.1 after stopping paracetamol 4 g daily taken for 30 days).

This is the first study to use the innovative WCE method to model the association between the duration, dose and recency of paracetamol exposure and COPD exacerbation. Our results suggest that there may be a complex association between paracetamol use and COPD exacerbation, with the effect differing according to these exposure variables. We found an increased risk of COPD exacerbation between 1 and 6 weeks after starting treatment and a lower risk in the first week after commencing paracetamol, suggesting the effect is not immediate. This observation may be consistent with the trend towards a gradual reduction in total antioxidant activity over 14 days observed in individuals who were administered maximum therapeutic doses of paracetamol (1 g four times a day) over this period [9].

These short-term and delayed effects of paracetamol are suggestive of an adaptive response of lung glutathione levels, which has been observed in previous studies with cigarette smoke exposure [27]. Lung epithelial lining fluid has a high concentration of glutathione, which protects against oxidants. The importance of antioxidants in the high oxygen environment of the lung may prevent glutathione levels from dipping below basal levels. Factors which disrupt this adaptive response may contribute to COPD [27]. In patients with existing COPD, if this impaired adaptive mechanism is replicated in lung glutathione depletion due to paracetamol exposure, it may explain the fluctuations in the risk of COPD exacerbation after initiation and discontinuation of paracetamol in our results.

Our observation that the weights switched from negative to positive 1 week post-initiation may explain the conflicting results found in studies of paracetamol and asthma [2–8, 28]. A study [17] found that people with a first diagnosis of COPD were more likely to have used paracetamol in the 30 days prior to diagnosis (any use within the past 30 days OR = 1.53, 95% CI = 1.32–1.78) but not between 31 and 365 days (OR = 1.09, 95% CI 0.93–1.27). However, that study did not report the prevalence of co-morbid arthritis, so it cannot be determined if differences in paracetamol use were due to differences in co-morbidity profiles. Osteoarthritis, a condition for which regular paracetamol use is commonly prescribed, is frequently co-morbid with COPD. A systematic review involving 14 studies assessing the prevalence of osteoarthritis in COPD found it ranged between 12 and 74%, with a weighted average of 36% [29]. In this study, the prevalence of co-morbid osteoarthritis was 70%, which was at the high end of the range.

One of the strengths of this study is our use of the DVA population database, which comprises a large representative population of elderly Australians. These data contain all medicine and hospitalization visits claimed by Australian DVA clients, including paracetamol obtained by prescription through the Pharmaceutical Benefits Scheme. Medicine use was determined through dispensing which limits potential response bias in recalling paracetamol use. Another strength of our study was the use of the WCE method that enables quantification of time-varying risk in a way that is potentially useful to patients and clinicians to make decisions. These WCE models have an advantage over classical time varying models since they are able to enumerate risk for a number of plausible patterns of medicine exposure and may help to highlight how treatment regimens may be optimised to balance benefits and risks. Due to the use of cumulative exposure and weighting by recency, the WCE method is able to account for differences between acute and chronic use. Finally, we used two different study designs to confirm the shape of the weight function and size of the time window.

There are some limitations to our study. During the time period of this study, DVA clients could also access paracetamol over the counter in pharmacies or supermarkets, which would not appear in the claims data, or by prescription. If no record of DVA status was recorded the prescription data would also be missing. Although most use would have been by prescription due to a subsidy by DVA, we may have underestimated exposure duration due to purchasing over the counter. Daily dose was not recorded and we assumed the maximum allowable dose and duration was a function of the number of tablets dispensed. Exposure may have been misclassified if the medicine was taken intermittently or at a lower daily dose. However, approximately two-thirds of our cohort were dispensed at least one prescription of 665 mg controlled release paracetamol tablets, which are recommended to be taken on a regular basis for osteoarthritis. COPD severity was not recorded so we defined severity by past use of corticosteroids and hospitalization for COPD, which may have led to misclassification. Our results may be influenced by “reverse causation”, where for example paracetamol may be initiated in the community for fever associated from a COPD exacerbation prior to a later hospital admission. A study of pain relief for COPD exacerbation found that 92% of patients experienced pain during an exacerbation compared with 58% during the stable phase (p < 0.001) and 30% received paracetamol in the emergency department, either alone or in combination [30]. However, the potential impact of “reverse causation” on our results is likely reduced given the significant proportion of chronic users, co-morbid osteoarthritis and use of controlled release paracetamol, which is unlikely to be used in this acute manner. Short-term use does not influence the results from longer exposure durations. We used ICD-10 codes for COPD hospitalization to define both the cohort and the outcome, which may have resulted in a loss of sensitivity [31]. However, since we excluded hospitalization for bronchitis, the outcome is more likely to represent true exacerbation and increase specificity. Lastly, it is possible that some residual confounding remained due to unmeasured confounders in our data such as environmental pollution, smoking status, and obesity [17, 18].

During the current COVID-19 pandemic, the role of glutathione in protection from severe disease has been postulated [32]. If there is a mechanism of action where glutathione depletion causes more severe disease, our results have implications for the cumulative dose and duration of paracetamol, which may be used for reducing fever in COVID-19 patients.

Our results show that understanding the complex relationship between exposure and risk can aid decisions on dosing for effective pain relief while minimising harm. By using the WCE method to model exposure, our study has found a complex relationship between paracetamol exposure and COPD exacerbation. The fluctuation in risk over time may explain some of the conflicting results in previous studies of paracetamol exposure and respiratory disease. One hypothesis is that this relationship may be due to initial depletion in pulmonary glutathione levels from paracetamol metabolism, followed by a rebound, which may warrant further investigation in future studies.

## Conclusions

This study adds to existing evidence that paracetamol use is associated with COPD. Our finding that the risk of hospitalisation for a COPD exacerbation fluctuated with time assist in explaining the conflicting results from previous studies regarding the potential impact of paracetamol with other respiratory diseases, particularly asthma. Cumulative paracetamol exposure may have a short-term increased risk 1 to 6 weeks after initiation or after stopping, suggesting that patients with COPD initiating or stopping regular high dose paracetamol should be monitored over this period. Beyond this period, there may be no long-term increase in risk from cumulative exposure after approximately 2 months.

Patients and doctors should be aware of the possible risk of COPD exacerbation with higher dose paracetamol 1 to 6 weeks after initiation or discontinuation, but no increased risk after 2 months.

## Supplementary Information

**Additional file 1: Figure S1.** Adjusted Hazard Ratios for different patterns of cumulative paracetamol dose and duration of exposure (compared to non-use) for 4 g daily. Dashed lines indicate confidence bands.

**Additional file 2: Figure S2.** Adjusted Hazard Ratios for past users compared to non-users, days after stopping paracetamol 4 g daily taken for 75 days or longer. Dashed lines indicate confidence bands.

**Additional file 3:** Supporting Information.

## Data Availability

The data that support the findings of this study are available from the Australian Government Department of Veterans’ Affairs but restrictions apply to the availability of these data, which were used under license for the current study, and so are not publicly available. Data are however available from the authors upon reasonable request and with permission of the Australian Government Department of Veterans’ Affairs.

## Abbreviations

ATC: Anatomical therapeutic chemical
COPD: Chronic obstructive pulmonary disease
DVA: Department of Veterans’ Affairs (Australian Government)
ICD-10-AM: International Classification of Disease, 10th Revision, Australian Modification
WCE: Weighted cumulative exposure
