# Supplementary figures and images for: The association between exacerbation of chronic obstructive pulmonary disease and timing of paracetamol use: a cohort study in elderly Australians
Source: Respir Res. 2022 Apr 5;23:80. doi: 10.1186/s12931-022-02010-z (PMC8979782; doi:10.1186/s12931-022-02010-z)

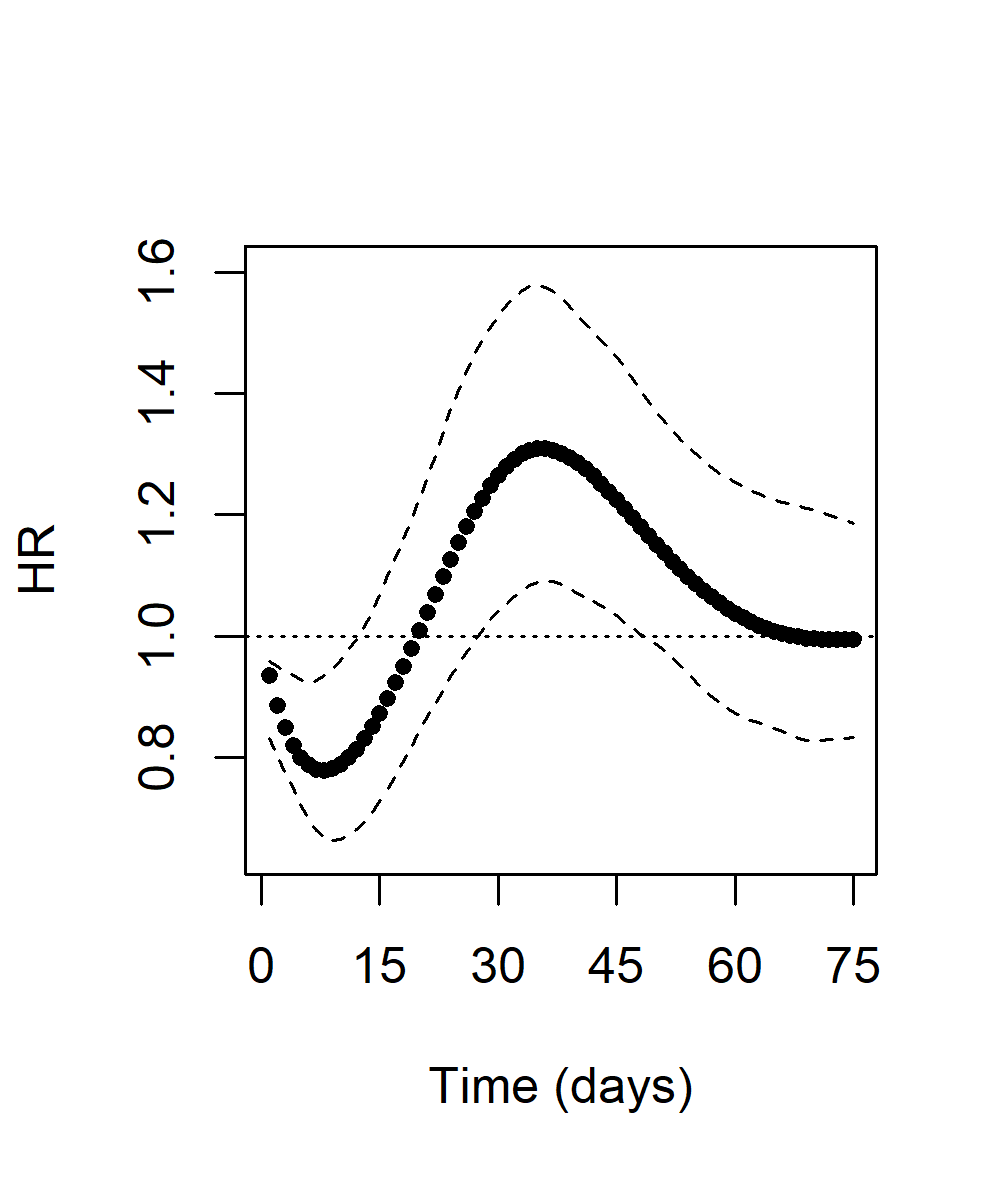

Supplement: Supplementary file 1 — Additional file 1: Figure S1. Adjusted Hazard Ratios for different patterns of cumulative paracetamol dose and duration of exposure (compared to non-use) for 4 g daily. Dashed lines indicate confidence bands. [file 12931_2022_2010_MOESM1_ESM.tif]

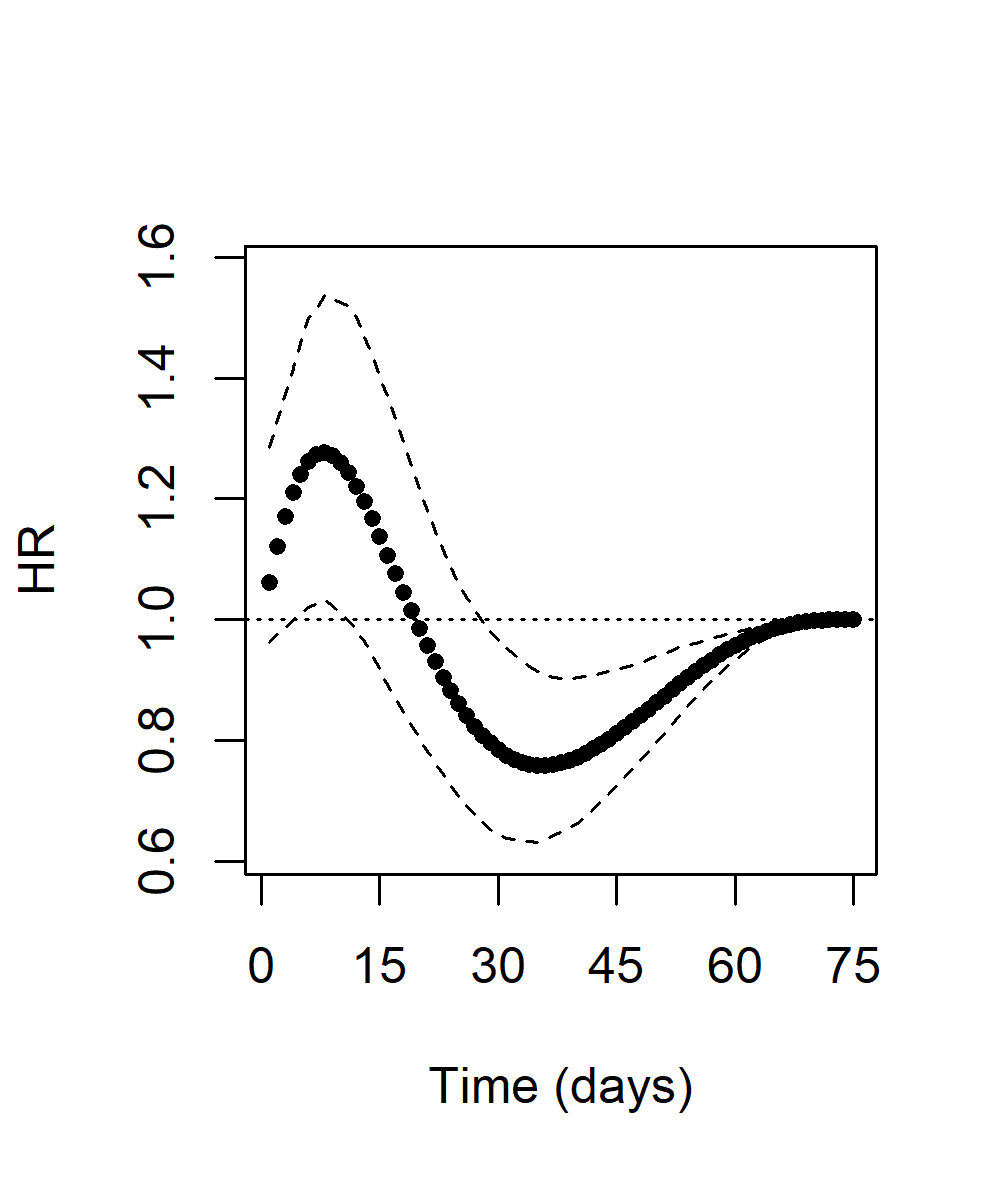

Supplement: Supplementary file 2 — Additional file 2: Figure S2. Adjusted Hazard Ratios for past users compared to non-users, days after stopping paracetamol 4 g daily taken for 75 days or longer. Dashed lines indicate confidence bands. [file 12931_2022_2010_MOESM2_ESM.tif]
