# Supplementary material for: The association between exacerbation of chronic obstructive pulmonary disease and timing of paracetamol use: a cohort study in elderly Australians
Source: Respir Res. 2022 Apr 5;23:80. doi: 10.1186/s12931-022-02010-z (PMC8979782; doi:10.1186/s12931-022-02010-z)
Supplement: Supplementary file 3 — Additional file 3: Supporting Information. [file 12931_2022_2010_MOESM3_ESM.docx]

**Additional file**

**List of Australia Pharmaceutical Benefits Scheme (PBS) paracetamol codes**

- 500g mg tablet (PBS code 10582Y,10585D,1746X,5196L,5224Y,8784H)
- modified release tablet 665g (PBS codes 10598T, 5343F, 8814X, 10796F, 10797G)
- Paracetamol + codeine combination (PBS code 10186D, 1215Y, 3316M, 4170L, 4171M, 4275B, 8785J)

**Comparison of WCE & conventional models in the cohort & nested case-control studies**

To compare the weighted cumulative exposure (WCE) and conventional models, Aikake Information Criterion (AIC) statistics were used to determine the specific weight function that represented the best fit to the data and to select the most appropriate time window. The weight function with the lowest AIC was considered to be the best fitting model, which was selected from splines with one to three knots and time windows of 15, 30, 45, 60, 75, 90 and 180 days. A difference of more than 4 points in the AIC indicated a better model fit^1^. 95% confidence intervals were generated by finding the 2.5^th^ and 97.5^th^ percentiles from 500 bootstrapped replicates.

For each model type, unadjusted and adjusted models were compared, with adjusted models giving a better fit according to the AIC. Therefore, the best fitting model was selected from the adjusted WCE and conventional models only.

In the cohort study, the WCE Cox model over 75 days (adjusted AIC 8463) represented a better fit compared with the best fitting conventional model, which defined exposure as the mean exposure during the previous 30 days (adjusted AIC 8470) (Table S1). In the nested case-control study, the conventional model with mean exposure in the 30 days before the index date had an AIC score within 1 point of the WCE model (adjusted AIC 1265) and therefore we could not distinguish between the fit of these two models. All four models in Table A1 had similar point estimates of risk above 1.0, however the width of the confidence intervals varied (survival models: conventional HR = 1.17, 95% CI = 0.95-1.44; WCE HR =1.27, 95% CI = 1.06-1.52; NCC models: conventional OR = 1.29, 95% CI = 0.99-1.69; WCE OR=1.03, 95% CI = 0.69-1.43). Since the WCE survival model had narrower confidence intervals for the weights than the NCC WCE model and had a lower AIC than the conventional survival model, it was selected as the best fitting model overall. Table S2 shows the odds ratio estimates for the covariates in the NCC model.

**References**

1. Abrahamowicz M, Beauchamp M-E, Sylvestre M-P. Comparison of alternative models for linking drug exposure with adverse effects. Stat Med 2012 (11-12);31:1014-30

**Table S1**

**Comparison of survival models and nested case-control (NCC) models**

| **Model** | **Exposure Pattern** | **Comparison** | **Adjusted Estimate**^1^ **(95% CI)** | **AIC** |
| --- | --- | --- | --- | --- |
| **Survival models** |  |  |  |  |
| Conventional | Total dose in the previous 30 days | Every 4 g increase per day | 1.17 (0.95, 1.44) | 8470^2^ |
| WCE | Current user of 4 g daily for 30 days^3^ | Non-users | 1.27 (1.06, 1.52) | 8463^4^ |
| **NCC models** |  |  |  |  |
| Conventional | Mean dose in 30 days before the index date | Every 4 g increase | 1.29 (0.99, 1.69) | 1265^5^ |
| WCE | Current user of 4 g daily for 30 days before the index date^3^ | Non-users | 1.03 (0.69, 1.43) | 1265^4^ |

^1^Hazard Ratio for Cox PH models; Odds Ratio for NCC models
^2^Best fitting conventional Cox PH model
^3^Exposure duration chosen for comparison with the best fitting conventional model
^4^AIC for best fitting WCE weight function with a time window of 75 days
^5^Best fitting conventional NCC model

**Table S2**

**Covariate estimates from the WCE NCC model**

| **Variable** | **Comparison** | **Adjusted Odds Ratio** **(95% CI)** | **p-value** |
| --- | --- | --- | --- |
| Statin use^1^ | Any use vs no use | 0.73 (0.59, 0.91) | 0.005 |
| Hypertension^2^ | Yes vs No | 0.88 (0.71, 1.10) | 0.27 |
| Congestive Heart Failure^2^ | Yes vs No | 1.33 (1.04, 1.72) | 0.03 |
| Diabetes^2^ | Yes vs No | 0.96 (0.68, 1.35) | 0.82 |
| Rx-Risk co-morbidity score^2,3^ | Every extra co-morbidity | 1.05 (1.01, 1.10) | 0.03 |
| Number of health services^4^ (log-transformed) | Every extra service | 1.40 (1.15, 1.70) | 0.0006 |

^1^In the 30 days before the index date ^2^Based on medication use in the 12 months before the index date
^3^Excluding hypertension, congestive heart failure, diabetes and COPD
^4^In the 12 months before the index date
